# Aspects of diastolic dysfunction in patients with new and recurrent depression

**DOI:** 10.1371/journal.pone.0228449

**Published:** 2020-01-31

**Authors:** Mariana Tudoran, Cristina Tudoran, Tudor Ciocarlie, Catalina Giurgi-Oncu

**Affiliations:** 1 Department VII Internal Medicine II, Discipline of Cardiology, University of Medicine and Pharmacy ʺVictor Babesʺ, Timisoara, Timis, Romania, County Clinical Emergency Hospital, Timisoara; 2 Neuroscience Department VIII, Discipline of Psychiatry, University of Medicine and Pharmacy ʺVictor Babesʺ, Timisoara, Timis, Romania, County Clinical Emergency Hospital, Timisoara; University of Palermo, ITALY

## Abstract

**Objective:**

The main objective of this study was to evidence the potential impact of the intensity, duration and recurrence of depression on the development of arterial stiffness (AS) leading to left ventricular hypertrophy (LVH) and diastolic dysfunction (DD) in patients with new onset depression (NOD) and recurrent depression (RD) in comparison to 33 control subjects without depression. Another aim was to identify potential predictive factors regarding the occurrence of diastolic dysfunction (DD).

**Methods:**

Our study group included 58 patients diagnosed with NOD and 128 diagnosed with RD, without any previously diagnosed significant heart diseases. The intensity of depression was evaluated by means of the Montgomery-Asberg Depression Rating Scale (MADRS). Assessment of pulse wave velocity (PWV), left ventricular mass index (LVMI) and echocardiographic parameters characterizing DD were performed for each patient.

**Results:**

The cardiology evaluations suggested an increased prevalence of AS in all patients, of significantly higher rate than in controls (p<0.001), which was statistically correlated with the severity and duration of depression. Another significant finding was an increased prevalence of DD (29.31% and 63.28%, respectively; p<0.001) correlated with the MADRS score, total duration and number of recurrences/relapses. The multivariate logistic regression analysis identified PWV, the intensity and duration of depression as significant predictive factors for the occurrence of DD.

**Conclusions:**

In our study, diastolic dysfunction was a common finding among patients with RD, but it was also noted, to a lesser extent, in those suffering with NOD. DD was associated with altered AS, and strongly correlated with the intensity and the duration of depressive symptoms. The two latter factors, together with an increased PWV, were strong predictors for the occurrence of DD.

## Introduction

In recent decades, Major Depressive Disorder (MDD) and Cardiovascular Diseases (CVD) are increasingly recognized as representing some of the most significant public health challenges. Numerous contemporary studies have found proof for the multiple connections that exist between the two pathologies [1, 2]. Existing data suggest that in patients without previous heart diseases, MDD is associated with a high risk of developing atherosclerosis and heart failure. On the other hand, in patients with preexisting coronary artery disease, arrhythmias or heart failure, comorbid MDD represents a negative prognosis influence, thus being considered an important risk factor [3, 4]. It is a well-recognized theory that depression is commonly associated with an unhealthy lifestyle, which results from a variety of factors, such as a poor diet and a sedentary lifestyle, which may, in turn, lead to obesity. Additionally, other unhealthy lifestyle choices, such as smoking, alcohol misuse, inconsistent treatment adherence, taken individually or in association with other potential individual risk factors, such as diabetes mellitus and dyslipidemia, may contribute to an early onset of atherosclerosis [5]. Moreover, findings from various clinical studies provide robust evidence on the complex relationship between MDD and CVD, as they appear to share common socio-demographic, psychosocial, and biological pathways. There are several hypothetical pathophysiological pathways shared by the two illnesses. One of these is represented by the dysregulation of the hypothalamic-pituitary-adrenal axis, which would result in an increased/prolonged release of cortisol and norepinephrine [6]. Together with sympathoadrenal hyperactivity and increased vasoconstrictive tone [6], this may induce systemic hypertension (SH), left ventricular hypertrophy (LVH) and diastolic dysfunction (DD). The imbalance of the autonomic nervous system represents another potential pathophysiological mechanism. Inflammation constitutes one of the most important contributing factors, owing to its association with increased levels of inflammatory markers, such as C-reactive protein (CRP), interleukin 1β and 6, tumor necrosis factor α, thus impacting on several cerebral neurotransmitter systems, including the serotonin, dopamine and glutamate pathways [7, 8]. Another significant mechanism implicated in the pathogenesis of psychiatric disorders is oxidative stress. It is known that the central nervous system is rich in phospholipids, which can be peroxided, generating reactive oxygen species (ROS), leading to protein oxidation, DNA and mitochondrial damage [9, 10]. In the last decade, several studies on animals suggest also, that cardiac oxidation could cause diastolic dysfunction even in the absence of hypertension or alteration of vascular elasticity [11]. Genetic factors may also play a significant role in the relationship between depression and CVD. There are assumptions that all these factors may induce the elevation of arterial stiffness (AS), followed by the development of SH and DD [12, 13, 14]. Several opinions in current literature suggest that there is a cumulative negative effect brought on by the exposure to repeated depressive episodes. These authors posit that there could be differences regarding cardiovascular impairment between patients with a long history of depression in the context of Recurrent Depression (RD) and those currently suffering from a first depressive episode [15, 16].

The main aim of this study is to document whether there are any differences regarding the severity of AS and DD alterations, in relation to the intensity and duration of depressive symptoms, between patients with new-onset depression (NOD) or RD, compared to subjects without comorbid depression. A secondary aim that we established for this study was to also identify the most important predictive factors responsible for the occurrence of these differences.

## Methods

The Ethics Committee of the County Clinical Emergency Hospital Timisoara approved this study and all patients signed informed consents.

***Study group*:** we selected a group of 176 patients (76 men and 110 women) who attended the outpatient mental health service of our general hospital, from 2014–2017, after being referred for depressive symptomatology. In the chronological order of their presentation, if they agreed to participate in the study, they were evaluated to see if they qualify for this analysis. ***The inclusion criteria were*: 1.** Patients with a first depressive episode (new-onset depression—NOD); **2.** patients with a new depressive episode in the context of RD (i.e., who reported suffering a minimum of one previous depressive episode); **3.** Lack of any antidepressant treatment at the moment of inclusion in the study; **4.** Adults aged under fifty-five years.

***Exclusion criteria*** were: **1.** previously diagnosed CVD (heart failure, moderate or severe hypertension, cerebrovascular, coronary, peripheral artery disease or arrhythmias); **2.** history of diabetes mellitus, dyslipidemia or other pathologies that could explain the occurrence of subclinical atherosclerosis; **3.** the presence of significant risk factors for atherosclerosis (including current smoking) and obesity IMC≥30 Kg/m^2^.

Considering the intensity of depressive symptoms, we proceeded to divide all patients into three subgroups:

- **group A:** fifty-eight patients with NOD;
- **group B:** one hundred and twenty-eight patients with RD;
- **group C:** thirty-three subjects, without depressive symptoms (MADRS scores 0 to 6), matched for age and which shared similar characteristics to our patients, selected from people attending the general practitioner’s outpatients service of our hospital in this period, served as the control group.

***Psychiatric evaluation*:** This was completed in the outpatient mental health service of our general hospital, for all patients that had been referred for depressive psychopathology between 2014–2017, in an effort to differentiate the presence of a NOD from a recurrence of previously diagnosed depressive illness. As per the international WHO classification guidelines, a depressive episode is characterized by the manifestation of a depressed mood that persists for a minimum of two weeks following a symptom free period of a minimum of 2 months, whereas a relapse signifies a reoccurrence of symptoms during the remission period, and before recovery had been achieved (within eight weeks). RD is defined as the occurrence of a new depressive episode after a recovery period of at least eight weeks.

The Montgomery-Asberg Depression Rating Scale (MADRS) was employed to evaluate the current intensity of depressive symptoms. This is a 10-item measure that assesses symptom variation in the past 7 days, while it is also used to evaluate the severity of depression based on the total score, with higher scores indicating a greater severity [16]. Each item is rated on a 7 point Likert scale, from 0 (no abnormality) to 6 (highest symptom intensity), with total scores varying from 0 to 60, and the following interpretation translating into intensities of current depressive symptoms: 0–6 = absence of depression; 7–19 = mild depression; 20–34 = moderate depression and 35–60 = severe depression. The MADRS has high interrater reliability, and it correlates significantly with scores of other standard scales for depression, such as the HAM-D [17].

***Cardiological evaluation*:** after a rigorous interview regarding personal medical history and a clinical examination, including repeated measurements of the right brachial systolic (SBPb) and diastolic blood pressure (DBPb), we determined the carotid to femoral pulse wave velocity (PWV) by using applanation tonometry (SphygmoCor, Atcor Medical) for each patient. Pressure waveforms were measured at the right common carotid and right common femoral arteries. In order to calculate the PWV, we measured the time needed for the arterial waveform to travel between two sites, along a vascular segment. A minimum of three determinations was performed for each patient. Subsequently, we proceeded by comparing these values with those determined in control patients.

The dimensions and function of cardiac cavities, the left ventricle (LV) mass index (LVMI) and the left atrial maximum volume index (LAVI) were evaluated by using a Siemens echocardiograph, in accordance to guidelines recommendations [18]. The same skilled operator completed these measurements in order to avoid inter-observer differences. LVH was defined as LVMI > 115 g/m^2^ for males and > 95 g/m^2^ for females. To assess the presence of DD, we recorded in pulsed Doppler, in apical 4-chamber view at the level of the mitral valve annulus, the mitral inflow and analyzed the peak early diastolic velocity (E), the late diastolic velocity (A), the E/A ratio, and the isovolumetric relaxation time (IVRT). Tissue Doppler imaging (TDI) was used for recording the mitral annulus early diastolic velocity (e’) and the late diastolic velocity at septal and lateral mitral annulus. Subsequently, an average E/e’ ratio was calculated. Tricuspid regurgitation velocity (TRV) was recorded by continuous-wave Doppler, from the apical window, at the level of the tricuspid valve. Type I DD was defined by an E/A ratio of ≤0.8 and E˂50cm/sec, while a type III DD was confirmed by an E/A ratio of over 2. In case of an E/A ratio≤0.8, but with an E of over 50 cm/sec, or if the E/A was between 0.8 and 2, a type II DD was considered, provided that a minimum of two of the following three criteria were present: an average E/e’>14, LAVI>34 mL/m^2^ and/or TRV>2.8 m/sec. In those cases, where only one criterion from the three previously mentioned was fulfilled, a type I DD was diagnosed [19].

***Data analysis*** was performed using SPSS v.25.0 (Statistical Package for the Social Sciences, Chicago, IL, USA) for Linux Mint 19. Continuous variables were presented as mean and standard deviations (SD) or as median and associated quartiles (Q1-25 percentage quartile, Q3-75 percentage quartile), while categorical data were presented as counts (percentages). The bias-corrected and accelerated (BCa) bootstrap interval (1000 bootstrap samples) was used to calculate the 95% confidence interval. We performed descriptive and inferential statistical analysis to summarize the characteristics of the study population. The results of the Shapiro-Wilk normality test showed a non-Gaussian distribution; therefore, we continued to use nonparametric tests. To evaluate the incidence of AS, LVH, and DD in groups, we applied the chi-squared analysis (χ2). To highlight the relationship between parameters characterizing AS, LVH and DD and the severity of depression, we performed the Spearman’s rank-order correlation. We used the Kruskal–Wallis H test, followed by post-hoc analysis with the Mann-Whitney U test with Bonferroni correction applied to compare the three groups (A, B, C). A forward stepwise multivariate logistic regression analysis was used to evaluate independent factors associated with the risk of developing DD. Akaike information criteria were used to determine the best regression model.

A p-value of less than 0.05 was considered to indicate a statistical significance.

## Results

We conducted this study on 186 patients, 76 men and 110 women, aged between 37 and 55 years, and a median age of 47.54 (±5.51) years, initially referred to our general hospital for a new depressive episode. Following a thorough mental health evaluation, fifty-eight patients were diagnosed with NOD, meaning that they were experiencing a first depressive episode. In the same vein, one hundred and twenty-eight patients were diagnosed as suffering with RD, with at least one previous episode of depression identified in their medical history. It was immediately established that none of these patients had a past diagnosis of CVD, diabetes mellitus or significant risk factors for atherosclerosis and none of them had been offered psychotropic medication for the current episode at the moment of inclusion in the study. We compared their results with data obtained in 33 age-matched subjects (13 men and 20 women). None of them showed altered AS, nor did anyone present DD. There were no statistically significant differences regarding the distribution of age between groups and controls, but the patients included in group A were younger than those from group B (p<0.001). The clinical and laboratory characteristics of study groups and control subjects are presented in Table 1.

**Table 1:** Results of clinical and laboratory determinations in patient groups.

Results of clinical and laboratory data, PWV and echocardiographic parameters	Group A: NOD 58 patients	Group B: RD 128 patients	Group C: Controls 33 patients	p		
				A-B	B-C	A-C
**Mean age** (years)	46 (42–49)	50 (44.25–53)	49 (45.5–52.5)	<0.001	0.929	0.124
**BMI** (kg/m^2^)	22 (22–25.3)	27 (25.52–28.1)	25.4 (24.55–26.15)	<0.001	<0.001	0.071
**LDL-cholesterol** (mg/dl)	109 (105–115)	117 (112–120)	96 (84.5–102)	<0.001	<0.001	<0.001
**CRP** (mg/dl)	2.15 (1.9–2.4)	3.45 (2–4.5)	1 (0.8–1.9)	<0.001	<0.001	<0.001
**Heart Rate (b/min)**	83 (76.75–88)	85 (78–88)	70 (68–73)	1	<0.001	<0.001
**SBPb (mmHg)**	135 (130–145)	130 (125–140)	120 (120–130)	0.189	<0.001	<0.001
**DBPb (mmHg)**	80 (70–90)	81.95 (77–90)	70 (70–75)	0.539	<0.001	<0.001
**Duration (months)**	1 (0.9–1.2)	13 (10–16)	-	<0.001	-	-
**MADRS**	31.5 (19–48)	28 (18–38)	2 (2–4)	0.116	<0.001	<0.001
**PWV** (m/sec)	7.9 (7.32–9.5)	10.6 (9.3–11.77)	6 (6–7)	<0.001	<0.001	<0.001
**LVMI** (g/m^2^)	95 (93–110)	100 (94.25–116)	93 (90–98)	<0.001	<0.001	1
**IVRT** (msec)	102 (99–104)	102 (95.25–109.75)	94 (91.5–96)	1	<0.001	<0.001
**VE** (m/sec)	0.7 (0.6–0.75)	0.63 (0.49–0.86)	0.87 (0.8–0.9)	0.938	<0.001	<0.001
**Ve’** (m/sec)	0.06 (0.04–0.07)	0.05 (0.035–0.07)	0.12 (0.09–0.14)	0.07	<0.001	<0.001
**E/A ratio**	1.31 (1.11–1.42)	0.85 (0.69–1.04)	1.29 (1.21–1.42)	<0.001	<0.001	0.215
**E/e´ ratio**	11.13 (10–12.58)	12.95 (9.65–16.75)	7.27 (6.16–8.66)	0.09	<0.001	<0.001
**LAVI mL/m**^**2**^	31 (31–32)	31 (29–36)	30 (30–31)	1	1	1
**TRV m/sec**	2.2 (2.1–2.5)	2.3 (1.9–2.8)	2 (2–2.1)	1	<0.001	<0.001

NOD–new-onset depression; RD–recurrent depression; BMI—body mass index; LDL–low density lipoproteins; CRP–C reactive protein; SBPb–brachial systolic blood pressure; DBPb–brachial diastolic blood pressure; MADRS—Montgomery-Asberg Depression Rating Scale; PWV–pulse wave velocity; LVMI–left ventricular mass index; IVRT–isovolumetric relaxation time; VE–velocity of peak early mitral inflow wave (E); Ve’–average peak early diastolic velocity of the mitral inflow in TDI; E/A—peak mitral inflow early (E) to late (A) diastolic velocities in pulsed Doppler; E/e’—early mitral inflow diastolic velocity E to average e’ velocity (E/e’) in TDI; LAVI—left atrial volume index; TRV -tTricuspid regurgitation velocity; (Kruskal Walis test).

***Group A*** contained a total of 58 patients, 25 men and 33 women, aged between 37 and 55 years, and a median age of 45.46 (± 4.52) years. The group included subjects currently diagnosed with NOD, and an intensity of depressive symptoms ranging from severe in 20 patients, to moderate in 23 patients and mild in 15 participants, with a median MADRS score of 31.5 (between 19–48).

Regarding AS, there were 16 patients (27.58%) with severe MDD and PWV >9 m/s, with a statistically significant difference when compared to controls and group B (Table 1). Also, we noted strong correlations between PWV and the severity of depressive episodes (r = 0.781, p<0.001). It should be noted that none of these patients presented LVH.

A pattern of type I DD, of altered relaxation, with an E/A ratio≤0.8 and an E<50cm/sec, was detected in 10 patients (17.24%). All other patients with an E/A ratio between 0.8 and 2, were further analyzed: type II DD was diagnosed in 4 subjects and type I in 3 participants, thus accounting for a total number of DD patients of 17, with a prevalence of 29.31%. An E/e’ of over 14 was detected in 12 of these subjects (70.58%). We determined moderate correlations between DD and the intensity of depression and PWV (Table 2).

**Table 2:** Relationship between DD and the studied parameters.

Parameter	Group A		Group B	
	r (95% CI)	p	r (95% CI)	p
**MADRS score**	r = 0.670 [0.500;0.813]	P<0.001	r = 0.670 [0.559;0.758]	p<0.001
**Age (years)**	r = 0.061 [-0.174;0.323]	p = 0.647	r = 0.585 [0.459;0.684]	p<0.001
**SBPb (mmHg)**	r = -0.054 [-0.325;0.221]	p = 0.688	r = 0.546 [0.405;0.661]	p<0.001
**DBPb(mmHg)**	r = -0.047 [-0.314;0.225]	p = 0.727	r = 0.654 [0.544;0.742]	p<0.001
**Duration of MDD**	r = 0.513 [0.288;0.691]	p = 0.001	r = 0.611 [0.479;0.710]	p<0.001
**Number of episodes**	-	-	r = 0.503 [0.376;0.632]	p<0.001
**PWV**	r = 0.577 [0.363;0.733]	p<0.001	r = 0.714 [0.605;0.804]	p<0.001
**LVMI**	r = -0.152 [-0.441;0.165]	p = 0.225	r = 0.374 [0.224;0.501]	p<0.001

MADRS—Montgomery-Asberg depression rating scale; SBPb–brachial systolic blood pressure; DBPb–brachial diastolic blood pressure; MDD–major depressive disorder; PWV–pulse wave velocity; LVMI–left ventricular mass index; CI–confidence interval; p–statistical signification; r–correlation; (Spearman’s correlation).

***Group B*** consisted of 128 patients, 51 men and 77 women, with ages ranging between 37 and 55, a mean age of 48.49 (±5.68), who had at least one previous episode of depression that was diagnosed between 6 to 27 months prior to the current evaluation. This patient group had a median mental illness duration of 13 (10–16) months, a group average of 2 previous depressive episodes (2 to 3) and a median MADRS score of 28 (between 18 to 38). According to this score, 40 patients had severe MDD, 47 suffered from moderate forms, and 41 patients reported mild intensities.

In this group, we repeatedly detected an increased PWV, of over 9 m/s, with the prevalence of altered AS being of 77,34% (99 patients), which represented a significant difference to controls (p<0.001) (Table 1). PWV was statistically strongly correlated with the severity and duration of the RD (r = 0.831 and r = 0.778, respectively; p<0.001), but only moderately associated with the number of depressive episodes (r = 0.675, p<0.001).

We detected LVH in 79 patients (61.71%). None of our patients had DD type III with E/A ratio of over 2. The prevalence of DD type I, when defined by a reduced E/A ratio ≤ 0.8 and E under 50 cm/sec, was 28.12% (36 patients). However, when we further analyzed all 92 patients with 0.8 <E/A ratio < 2, we could document a typical pattern in 47 patients (36.71%), mostly in those with mild and moderate depression. We diagnosed DD type II in 39 patients and DD type I in 6, the total number of patients with this type of DD being 42 and an overall DD prevalence of 63.28% (81 patients). We identified an elevated E/e’ ratio of over 14 in 61 patients (47.65%). We highlighted statistically significant correlations between the E/e’ ratio and the severity and duration of the depressive illness, as well as with the PWV, and moderate associations with LVMI and the number of episodes.

Multivariate logistic regression analysis was used to evaluate the independent predictor factors for the development of DD. The regression model was built based on the stepwise forward method, and Akaike information criteria were used to appreciate the best model (Table 3). We identified the most significant predictive factors for the occurrence of DD in all depressive patient (Table 3). In the whole group, our results highlighted that the severity of AS expressed by PWV (2-fold), the intensity and duration of depression, MADRS (1.07-fold) and duration of depression (1.15-fold), increase the risk of developing DD.

**Table 3:** Multivariate logistic regression analysis for the presence of diastolic dysfunction.

Factor	β	±SE	p	Odds ratio	95% Odds ratio
**PWV**	0.695	0.275	0.012	2.005	1.169–3.439
**Duration of depression**	0.147	0.065	0.025	1.158	1.019–1.317
**MADRS**	0.071	0.026	0.007	1.073	1.020–1.130

PWV–pulse wave velocity; MADRS—Montgomery-Asberg Depression Rating Scale; β –regression coefficient; SE–standard error; statistical method: multivariate stepwise linear regression (Akaike information criteria).

## Discussions

Our study analyzed potential significant associations between the intensity of depressive episodes in a group of 58 patients with NOD versus 128 patients with RD, who were evaluated in terms of alterations of AS and the existence and severity of LVH and DD, while taking into account the severity of depressive episodes. For RD patients, the duration and number of previous depressive episodes were also taken into consideration. The results were compared with data obtained in an age-matched control group.

In both patient groups, we repeatedly noted AS alteration, as expressed by elevated levels of PWV, of over 9 m/s, when compared with controls. The prevalence of increased PWV was of 27.58% in patients with NOD and of 77.34% in those with RD, with significant differences between them, and also when compared to controls (p<0.001). However, this finding was more frequent in patients with severe or moderate forms of RD, in comparison with those reporting milder symptom intensities. In group B, PWV was statistically strongly correlated with the duration and severity of depression, but the correlation with the length of RD was the strongest. The association with the number of depressive episodes was moderately statistically significant.

The association between MDD and early-onset atherosclerosis was analyzed in several studies. For example, Smith and Singer [5] debated the role of AS in the bidirectional relationship between depression and cardiovascular comorbidity in patients with MDD. In the Maastricht study, Onete et al. [13] evaluated MDD by using the Mini-International Neuropsychiatric Interview (MINI) and the Patient Health Questionnaire-9 (PHQ-9). They studied the association with AS by determining PWV in a cohort of 2757 participants, and found that MDD and depressive symptoms, as well as AS were higher among middle-aged men, and to a lesser extent, in women. Similar results were reported by Van Sloten et al. in the AGES-Reykjavik Study Artery Research [12]. Khandoker et al. analyzed the increased incidence of AS, as assessed solely by the PWV or by PWV and Fingertip Photo-Plethysmography in smaller groups of depressed patients with suicidal intention, but without comorbid CVD [20]. Other results of recent meta-analyses of prospective cohort studies, conducted on subjects that were initially without CVD, indicate that depression was associated with a 30% increased risk of future coronary events [21, 22].

As mentioned in our results, LVH and DD were frequently diagnosed in patients with RD, especially in those with severe and moderate forms. The incidence of DD was higher, when assessed by means of TDI (66.4%), in comparison with pulsed Doppler (38.28%). There were statistically significant differences between the type of DD in the two groups (p<0.001), namely DD type II was more frequent in patients with RD, being significantly correlated with PWV and with the intensity of current depressive symptoms, followed by the overall duration of RD.

New echocardiographic techniques like strain and strain rate promise to offer a superior evaluation of the systolic and diastolic function [23]. We performed this analysis only in a limited number of patients, mostly in those with NOD. Left ventricular global longitudinal strain was within normal limits in all cases, but referring to left atrial global strain, we obtained various patterns, with alterations of the reservoir, conduit or booster pump function, often combined, but the number of determinations was not sufficient to obtain statistically significant data. This aspect represents a limitation of our study.

The negative impact of MDD on the evolution of heart failure has been established previously. Recently, Feola et al. analyzed the connection between MDD, cognitive impairment and specific objective parameters characterizing heart failure (BNP, ejection fraction, cardiac output, etc.) in a group of 303 patients hospitalized for congestive heart failure [24]. Other authors debated the influence of MDD on the occurrence and evolution of heart failure. For example, Gustad et al. [15] analyzed the progress of 62 567 adults of both genders, from the second wave of the Nord-Trøndelag Health Study (HUNT 2, 1995–1997, Norway), which were initially free of heart failure, by following their evolution longitudinally. Thus, they found an increased incidence of heart failure in depressed patients, which was associated with elevated mortality. Similarly, Ogilvie [5] examined the relationship between psychological status and incidence of heart failure in 6782 individuals from the Multi-Ethnic Study of Atherosclerosis and reported that depressive symptoms were associated with an increased risk of heart failure. In a recent study, Wang et al. [25] also analyzed these aspects in a smaller group and concluded that MDD is a prevalent and independent predictor of mortality in patients with diabetes mellitus and heart failure.

Most existing studies have focused either on the prevalence of AS or heart failure in patients with MDD or on the impact of depression on the evolution of coronary artery disease or heart failure [21, 25]. In our study, we attempted to evidence, in patients with NOD and RD, the increased prevalence of AS alterations and of DD, when compared to controls. We suggested that the intensity of depressive symptoms and, in the case of RD, the duration and number of depressive episodes, could be directly related to the prevalence and severity of DD. Another aim of our study was to identify the main factors that could be responsible for the occurrence of DD. The multivariate logistic regression analysis identified multiple significant predictive factors. Firstly, a pronounced intensity of current depressive symptoms, as evaluated by the MADRS score, was associated with AS alterations with increased PWV. Besides these factors, in patients with RD, the overall illness duration had an important influence on the occurrence of DD. Consequently, our results appear to be supporting existing data in this research field, emphasizing the significant negative role held by the severity and duration of a comorbid depressive illness on the genesis and progression of subclinical atherosclerosis, LVH, DD and, ultimately, patent heart failure.

## Conclusion

In our study, diastolic dysfunction was a common finding among patients with recurrent depression, but was also noted in those with new-onset depression. DD was associated with subclinical atherosclerosis, as expressed by altered AS, and appeared to be strongly correlated with the intensity and duration of depressive symptoms. The last two factors, together with an increased PWV, appear to be strong predictors for the occurrence of DD.
